# Moderate SMFs attenuate bone loss in mice by promoting directional osteogenic differentiation of BMSCs

**DOI:** 10.1186/s13287-020-02004-y

**Published:** 2020-11-16

**Authors:** Guilin Chen, Yujuan Zhuo, Bo Tao, Qian Liu, Wenlong Shang, Yinxiu Li, Yuhong Wang, Yanli Li, Lei Zhang, Yanwen Fang, Xin Zhang, Zhicai Fang, Ying Yu

**Affiliations:** 1 Department of Pharmacology, Tianjin Key Laboratory of Inflammatory Biology, The Province and Ministry Co-sponsored Collaborative Innovation Center for Medical Epigenetics, School of Basic Medical Sciences, Tianjin Medical University, Tianjin, 300070 China; 2 Department of Orthopedics, Tianjin Medical University General Hospital, Tianjin, 300070 China; 3 High Magnetic Field Laboratory, Key Laboratory of High Magnetic Field and Ion Beam Physical Biology, Hefei Institutes of Physical Science, Chinese Academy of Sciences, Hefei, 230031 China; 4 Heye Health Industrial Research Institute of Zhejiang Heye Health Technology, Anji, 313300 Zhejiang China

**Keywords:** Bone marrow-derived mesenchymal stem cell, Static magnetic field, Osteoporosis, Osteogenic differentiation, Magnetic therapy

## Abstract

**Background:**

Osteoporosis is a common metabolic bone disease without effective treatment. Bone marrow-derived mesenchymal stem cells (BMSCs) have the potential to differentiate into multiple cell types. Increased adipogenic differentiation or reduced osteogenic differentiation of BMSCs might lead to osteoporosis. Whether static magnetic fields (SMFs) might influence the adipo-osteogenic differentiation balance of BMSCs remains unknown.

**Methods:**

The effects of SMFs on lineage differentiation of BMSCs and development of osteoporosis were determined by various biochemical (RT-PCR and Western blot), morphological (staining and optical microscopy), and micro-CT assays. Bioinformatics analysis was also used to explore the signaling pathways.

**Results:**

In this study, we found that SMFs (0.2–0.6 T) inhibited the adipogenic differentiation of BMSCs but promoted their osteoblastic differentiation in an intensity-dependent manner. Whole genomic RNA-seq and bioinformatics analysis revealed that SMF (0.6 T) decreased the PPARγ-mediated gene expression but increased the RUNX2-mediated gene transcription in BMSCs. Moreover, SMFs markedly alleviated bone mass loss induced by either dexamethasone or all-trans retinoic acid in mice.

**Conclusions:**

Taken together, our results suggested that SMF-based magnetotherapy might serve as an adjunctive therapeutic option for patients with osteoporosis.

**Supplementary information:**

**Supplementary information** accompanies this paper at 10.1186/s13287-020-02004-y.

## Background

Osteoporosis is a common bone and metabolic disease, characterized by reduced bone formation and increased fat accumulation in the bone marrow space, leading to decreased bone mineral density (BMD) and increased bone fractures [1]. As such, it is reported to be responsible for more than 1.5 million fractures worldwide annually [2]. Both adipose-derived stem cells (ASCs) and bone marrow-derived mesenchymal stem cells (BMSCs) are multipotent progenitor cells with the potential to differentiate into various mature cell types, including osteoblasts, chondrocytes, and adipocytes [3, 4], which have been utilized extensively in the research field of bone tissue regeneration. Despite higher proliferative capacity for ASCs, BMSCs display more potential for osteogenic and chondrogenic differentiation [5, 6]. Disrupting the dynamic balance between adipogenic and osteogenic differentiation of BMSCs, such as increased differentiation toward adipocytes or reduced differentiation toward osteoblasts, has been shown to cause osteoporosis and other bone and metabolic diseases [7, 8]. As pleiotropic drugs, metformin and rapamycin have been reported to promote bone generation and improve bone density by increasing osteogenic differentiation potential of BMSCs [9, 10]. Moreover, physical activities have also been found to induce mobilization of stem cells and promote bone regeneration [11, 12]. However, the mechanisms behind the fine-tuned regulation of the commitment of the BMSC lineage to differentiate to osteoblasts versus adipocytes remain elusive.

Humans are exposed to the naturally occurring magnetic fields of the earth. Lack of exposure to natural magnetic fields, such as staying in space, has been reported to cause insomnia, fatigue, depression, and increased predisposition for osteoporosis in humans [13], suggesting that the magnetic field might be beneficial for physiological function and human health. Accumulated evidence has shown that static magnetic fields (SMFs) might act on a variety of potential targeting organs and tissues, exerting favorable physiological effects on many biological systems, including suppression of inflammatory reactions [14], reduction of edema formation [15], improvement of microcirculation and blood flow [16, 17], relief of osteoarthritis-induced pain [18], and facilitation of wound healing [19]. Therefore, magnetotherapy has been officially approved by the US Food and Drug Administration (FDA) for the treatment of pain and edema in superficial soft tissues for orthopedic applications [20]. Moreover, moderate-intensity SMF has been shown to enhance repair after cartilage damage and accelerate the formation of new bone tissue in rat models, by promoting extracellular matrix deposition [21, 22]. SMF treatment has been reported to increase BMD of osteoporotic lumbar vertebrae in ovariectomized rats [23] and prevent the architectural deterioration and strength reduction of bones in streptozotocin-treated diabetic rats by enhancing osteogenic differentiation [24]. These observations strongly suggest that SMF might help prevent and treat osteoporosis. However, whether SMFs might participate in the reciprocal regulation between adipocyte and osteoblast differentiation of BMSCs and the subsequent control of the adipo-osteogenic balance remains to be determined.

In the present study, we stimulated BMSCs during adipogenic and osteogenic differentiation with different intensities of SMFs (0 T, 0.2 T, 0.4 T, and 0.6 T), and found SMFs promoted osteoblastic differentiation, but inhibited adipogenic differentiation of BMSCs in an intensity-dependent manner. Whole genomic RNA-seq analysis demonstrated SMFs decreased PPARγ-driven gene expression while increased RUNX2-driven gene transcription in BMSCs. Moreover, SMFs markedly alleviated bone mass loss induced by either dexamethasone (Dex) or all-trans retinoic acid (ATRA) in mice. Thus, our study reveals moderate intensity SMFs may serve as an adjunctive therapeutic option for osteoporosis.

## Methods

### Animals

Wild-type C57BL/6J mice were purchased from GemPharmatech Co. Ltd. and were housed in specific pathogen-free animal facilities of the Tianjin Medical University. Accordingly, 8–10-week-old male mice were used in this study. All animal experiments were performed in accordance with the Guidelines of the Institutional Animal Care and Use Committee of Tianjin Medical University.

### Reagents

CD29-APC, CD105-PE-Cy7, Sca1-FITC, and c-kit-APC antibodies were purchased from Thermo Fisher Scientific (eBioscience), while CD44-Percp5.5, CD90-BV421, CD34-PE, CD45-APC, and CD11b-FITC antibodies were obtained from Biolegend. Oil red O (ORO), alizarin red S (ALS), all-trans retinoic acid (ATRA), dexamethasone (Dex), and mineral oil were purchased from Sigma-Aldrich.

### Moderate SMF exposure system

**Fig. 1:**
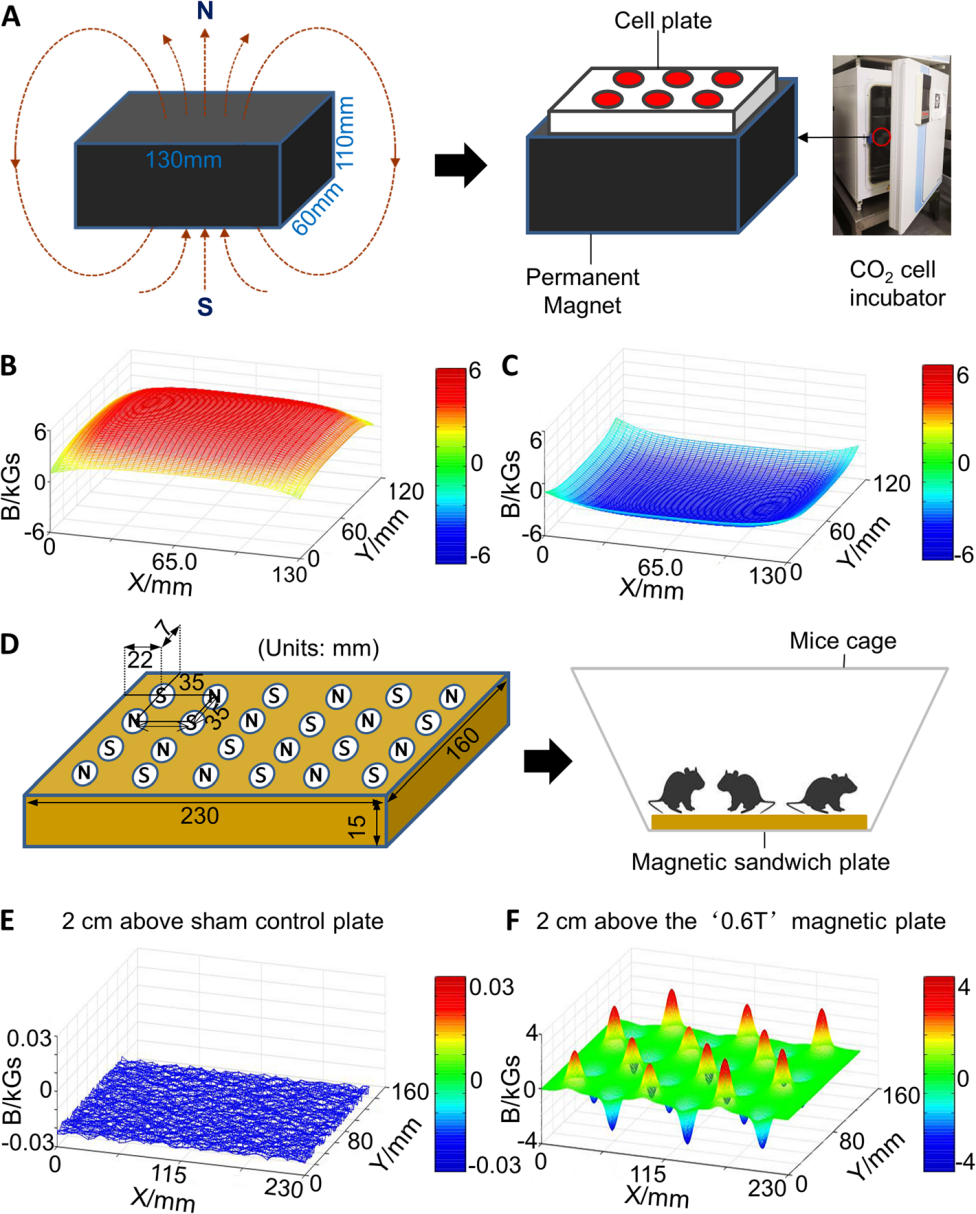
Schematic diagram of the static magnetic field (SMF) systems. **a** A permanent magnet assembled beneath the cell plates to generate an SMF environment. **b**, **c** Intensity distribution of SMF of the 130 × 110 × 60 mm magnet (0.6 T shown here) for the cell exposure experiments. **d** The magnetic or nonmagnetic sandwich plates (230 × 130 × 15 mm), in which 24 magnets (10 mm diameter and 15 mm thickness) were inserted with alternating magnetic poles (north or south; N or S) facing up, were placed beneath the cages in which mice were housed. **e–f** The distribution of SMF intensity of the 230 × 130 × 15 mm control magnetic equipment (0 T; **e**) and 0.6 T magnetic sandwich plate (**f**) used for the animal study

For the creation of the SMF exposure system, gradient permanent neodymium magnets (130 × 110 × 60 mm) were assembled beneath the cell plates to expose the cultures to north fields (N; Fig. 1a). The distribution of the representative SMF (0.6 T), measured using a digital Tesla meter (HT20), was shown in Fig. 1b, c. For mouse exposure, the magnetic or nonmagnetic sandwich plates (230 × 130 × 15 mm), in which 24 magnets (10 mm diameter and 15 mm thickness) were inserted with alternating magnetic poles (north or south; N or S) facing up, were placed beneath the mouse cages and separated by spaced holes (Fig. 1d) as previously reported [19, 25]. The SMF intensity distribution of control and 0.6 T magnetic sandwich plates were shown in Fig. 1e and Fig. 1f, respectively, as measured by a space magnetic field measuring instrument (FE-2100RD).

### Cell culture

BMSCs were isolated from femoral bones of male mice (4–6 week-old), as previously reported [26, 27]. At passage 3, BMSCs were subjected to purification by flow cytometry through the identification of CD29, CD44, CD90, CD105, and Sca-1 positive surface markers and CD11b, CD34, CD45, and c-kit negative surface markers, as shown in sFig. 1. Then, BMSCs were subjected to adipogenic and osteogenic differentiation using the respective induction media.

### Adipogenic differentiation assay

Isolated BMSCs were maintained in α-MEM containing 10% fetal bovine serum (16000-044, Gibco, USA), 100 U/mL penicillin, and 0.1 mg/mL streptomycin (C125CS, NCM Biotech). For adipogenic differentiation, BMSCs were grown in basal medium supplemented with 1 μM Dex (Sigma-Aldrich), 0.5 mM 3-isobutyl-1-methylxanthine (Sigma-Aldrich), 10 μg/mL insulin (Sigma-Aldrich), and 1 μM rosiglitazone (Sigma-Aldrich) for 14 days. The culture medium was replaced every other day. Fat droplet formation was visualized by ORO staining and quantified using the Image J software.

### Osteogenic differentiation assay

BMSCs were grown in basal medium until appropriate cell confluence (approximately 70%). For osteogenic differentiation, BMSCs were cultured in osteogenic induction medium containing 50 mM ascorbate-2-phosphate, 0.1 mM dexamethasone, and 10 mM β-glycerol phosphate for 21 days. The culture medium was changed every third day. After osteogenic differentiation for 3 days, cultured BMSCs were fixed with 70% (v/v) ethanol and incubated in 0.25% (w/v) naphthol AS-BI phosphate solution and 0.75% (w/v) Fast Blue BB in 0.1 M Tris buffer before alkaline phosphatase (ALP) staining. A commercial ALP activity kit (Sigma-Aldrich) was used to assess the ALP activity of differentiated cells according to the manufacturer’s instructions, following normalization by total cell protein. At the end of differentiation, alizarin red S (ALS) staining was performed to evaluate the mineralization of the cell matrix.

### Histological analysis

Paraffin-embedded femurs were sectioned (5 μm), deparaffinized, and then stained with hematoxylin and eosin (H&E) for morphological analysis. For immunohistochemistry staining, deparaffinized and dehydrated tissue sections were re-hydrated before being subjected to antigen retrieval, and then blocked with diluted normal serum for at least 1–1.5 h at 25 °C to eliminate nonspecific binding. The slides were incubated with primary antibodies against osteocalcin (OCN) (1:300; Abcam) overnight at 4 °C. After careful washing, the sections were incubated with horseradish peroxidase conjugates to detect positive signals, followed by counterstaining with hematoxylin (Sigma-Aldrich). Slides incubated with polyclonal rabbit IgG (Abcam) served as negative controls. Pictures were captured and monitored using a Leica Microsystems microscope (Leica Microsystems Ltd.), while the ImageJ software (National Institutes of Health) was used to analyze the number or area of adipocytes and osteoblasts.

### Real-time quantitative PCR

Total mRNA was extracted from BMSCs grown in adipogenic or osteogenic medium using TRIzol reagent (Invitrogen) and reverse-transcribed to cDNA using a Reverse Transcription Reagent kit (Takara Bio Inc.) according to the manufacturer’s protocol. The resulting cDNA was amplified using the Real-Time PCR system (LightCycler 480 II, Roche) with 40 cycles. Sequences of primers for target genes are presented in Supplementary Table 1. The mRNA levels of the specific genes were normalized to that of a reference gene (β-actin) within the samples.

### Western blotting

After being cultured in adipogenic or osteogenic medium, BMSCs were harvested and lysed with RIPA (Solarbio). Protein concentrations were quantified using a BCA Protein Assay Kit (Thermo Fisher Scientific). Equal amounts of protein were denatured and resolved by 10% sodium dodecyl sulfate-polyacrylamide gel electrophoresis, transferred to polyvinylidene fluoride membranes (Millipore), incubated with 5% skimmed milk (Biofath) for 1–1.5 h at 25 °C, and then incubated overnight at 4 °C with primary antibodies. Primary antibodies were diluted as follows: PPARγ (1:500; Santa Cruz Biotechnology), Fabp4 (1:1000; Abcam), Runx2 (1:1000; Cell Signaling Technology), and ALP (1:1000; Santa Cruz Biotechnology). β-Actin (1:5000; Cell Signaling Technology) or α-Tubulin (1:10,000; Sungene Biotech) was used as loading control. Consecutively, the membranes were incubated with horseradish peroxidase-conjugated secondary antibodies (1:2000; Cell Signaling Technology) dissolved in blocking buffer for 2 h at 25 °C. Blots were detected using an enhanced chemiluminescent reagent kit (Thermo Fisher Scientific).

### RNA-seq

BMSCs plated in 10 cm dishes were exposed on a control iron plate (0 T) or a magnetic plate (0.6 T) for 48 h respectively. Then, BMSCs were washed by cold PBS for 3 times and then lysed by using Trizol at room temperature. Samples were sent to Novogene on dry ice for library preparation and sequencing. A total amount of 3 mg RNA per sample was used as input material for the RNA sample preparations. Sequencing libraries were generated using NEBNext UltraTM RNA Library Prep it for Illumina (NEB, USA) following manufacturer’s recommendations and index codes were added to attribute sequences to each sample. The clustering of the index-coded samples was performed on a cBot Cluster Generation System using TruSeq PE Cluster Kit v3-cBot-HS (Illumia) according to the manufacturer’s instructions. After cluster generation, the library preparations were sequenced on an Illumina platform and 150 bp paired-end reads were generated. R software version GSEA and package fgsea were used for cellular pathway analysis including Hallmark gene sets and GO gene sets.

### Microcomputed tomography (micro-CT) scan

Femurs of the same side were dissected from mice treated with ATRA for 21 days [28] or treated with Dex for 3 months [29] and fixed with 4% paraformaldehyde for more than 24 h. Then, the samples were scanned and analyzed using a micro-CT System (Skyscan 1172, Bruker) with a high resolution (voltage 50 kV; current 201 μA; resolution 12 mm/pixel), as reported [30]. The NRecon image reconstruction software (Bruker), CTVol 3D model visualization software (Bruker), and CTAn data analysis software (Bruker) were used to analyze the parameters of the trabecular bones. For distal femurs, regions of interest were selected for the analysis of BMD, trabecular bone volume per tissue volume (Tb.BV/TV), trabecular separation (Tb.Sp), trabecular number (Tb.N), and trabecular thickness (Tb.Th).

### Statistical analysis

Data were analyzed using GraphPad Prism version 6.0 and are presented as the mean ± standard error of the mean. Two-tailed unpaired Student’s *t* test or one-way ANOVA with Bonferroni post hoc analyses were applied when appropriate for multiple comparisons. A value of *P* < 0.05 was considered statistically significant.

## Results

### Moderate SMFs inhibited adipogenic differentiation of murine BMSCs

**Fig. 2:**
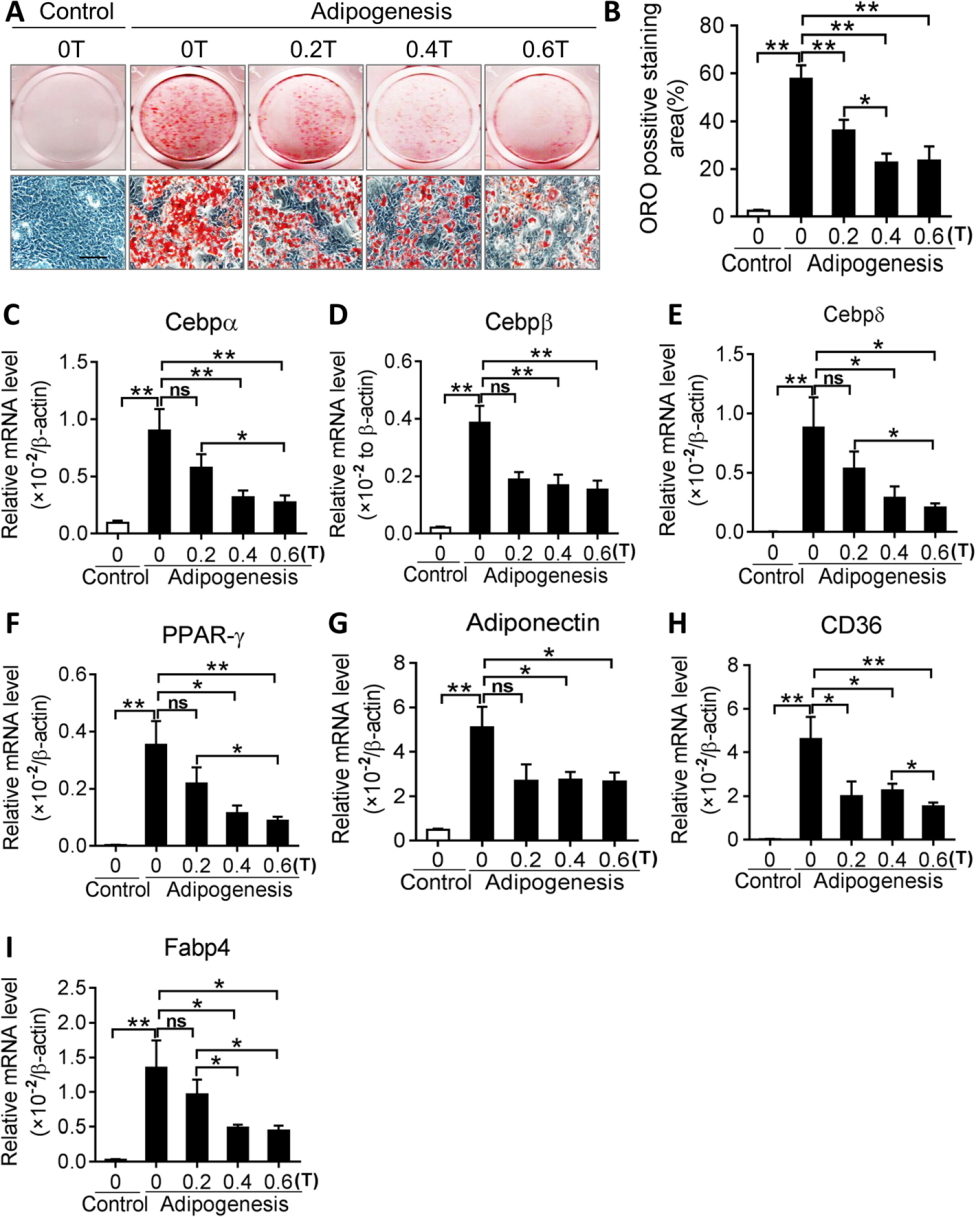
SMF suppresses adipogenic differentiation of bone marrow-derived mesenchymal stem cells (BMSCs). **a** Representative images of oil red O (ORO) staining of lipids in BMSCs cultured in adipogenic medium and treated with gradient SMFs (0, 0.2, 0.4, and 0.6 T) for 14 days. Scale bar, 100 μm. **b** Quantification of the ORO-positive staining area. *P < 0.05, **P < 0.01, as indicated. *n* = 4 per group. **c**–**i** Expression of adipogenic transcription factors (**c**–**f**) and marker genes (**g**–**i**) in BMSCs grown in adipogenesis induction medium and treated with gradient SMFs. *P < 0.05, **P < 0.01, as indicated. *n* = 8–10 per group. ns=not significant

To investigate the effect of SMFs on the adipogenic differentiation of BMSCs, BMSCs grown in adipogenic medium were exposed to different intensities of moderate SMFs (0, 0.2, 0.4, and 0.6 T). ORO staining showed that SMFs significantly inhibited lipid-droplet formation in BMSCs in adipogenic medium in an intensity-dependent manner (Fig. 2a, b). The expression of adipogenic transcription factors, such as CCAAT/enhancer-binding protein α, β, and δ (*Cebpα*, *Cebpβ*, and *Cebpδ*), was shown to be lower in SMF-stimulated BMSCs than those in control cells (Fig. 2c–e). Moreover, the mRNA levels of *Pparγ* adipogenic marker and those of its downstream targets, adiponectin, cluster of differentiation 36 (*Cd36*), and fatty acid binding protein 4 (*Fabp4*) (Fig. 2f–i), were demonstrated to be markedly decreased in SMFs-treated BMSCs compared with iron control (0 T). In contrast, high intensity SMF (0.6 T) displayed a more apparent inhibitory effect on BMSC differentiation toward adipocytes than lower intensities (0.2 and 0.4 T; Fig. 2a–f).

### Moderate SMFs suppressed PPARγ-mediated signaling in BMSCs upon adipogenic differentiation

**Fig. 3:**
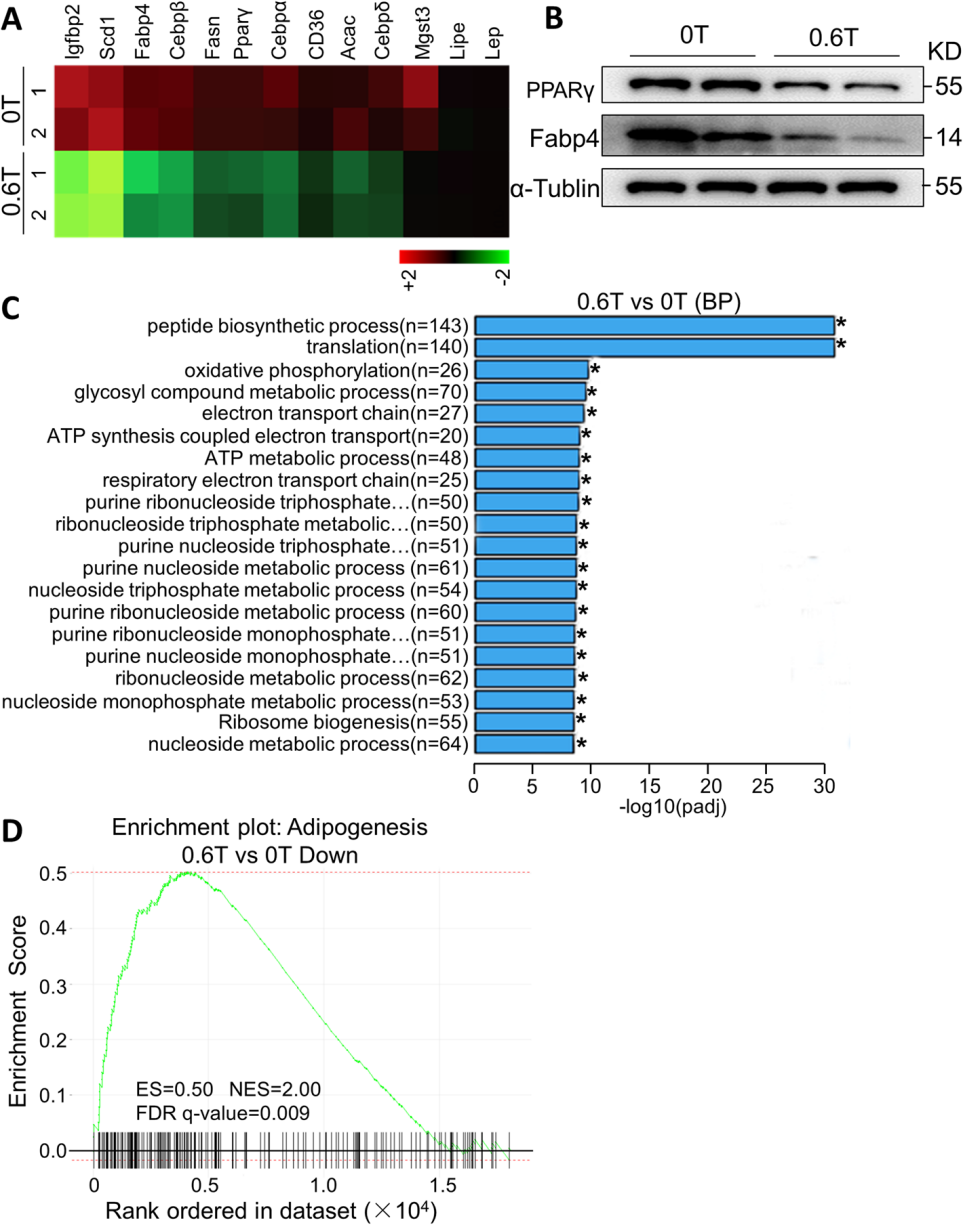
SMF inhibits peroxisome proliferator-activated receptor γ (PPARγ)-mediated signaling in BMSCs cultured in adipogenic medium. **a** Heatmap of the mRNA expression of downregulated adipogenic genes, as detected by deep sequencing, in BMSCs grown in adipogenic medium and stimulated with 0.6 T SMF for 48 h (fold change > 2, false discovery rate < 0.20). **b** Western blot analysis of PPARγ and FABP4 in SMF-exposed BMSCs cultured in adipogenic medium. **c** Gene Ontology (GO) pathway enrichment analyses of significantly downregulated genes in SMF-exposed BMSCs cultured in adipogenic medium. Top 20 enriched pathways are shown.*P<0.05. **d** Gene set enrichment analysis (GSEA) indicates that PPARγ-positively correlated genes are significantly reduced in SMF-exposed BMSCs compared with those in control cells

RNA profile analysis also revealed that some adipogenic transcription factors (*Cebpα*, *Cebpβ*, *Cebpδ*, and *Pparγ*) and adipocyte marker genes (*CD36*, *Fabp4*, *Igfbp2*, *Scd1*, *Fasn*, *Lpi*, *Mgst3*, and *Lep*) were downregulated in 0.6 T SMF-stimulated BMSCs compared with those in unchallenged BMSCs (Fig. 3a). The downregulation of the *Pparγ* and *Fabp4* genes was further validated in SMF-stimulated BMSCs by western blot analysis (Fig. 3b). To correlate the differentially expressed genes with biological functions, we analyzed the functional bias of the differentially expressed genes according to Gene Ontology (GO) enrichment. Our results revealed that SMF altered many metabolic processes in BMSCs, including peptide biosynthetic process, translation, ATP metabolic process, purine ribonucleoside metabolic process, and nucleoside metabolic process (Fig. 3c). Gene-set enrichment analysis (GSEA) showed that PPARγ-positively correlated genes were remarkably enriched in iron control-treated BMSCs, compared with those in SMF-exposed cells (false discovery rate (FDR) *q* value = 0.009) (Fig. 3d).

### Moderate SMFs promoted osteogenic differentiation of murine BMSCs

**Fig. 4:**
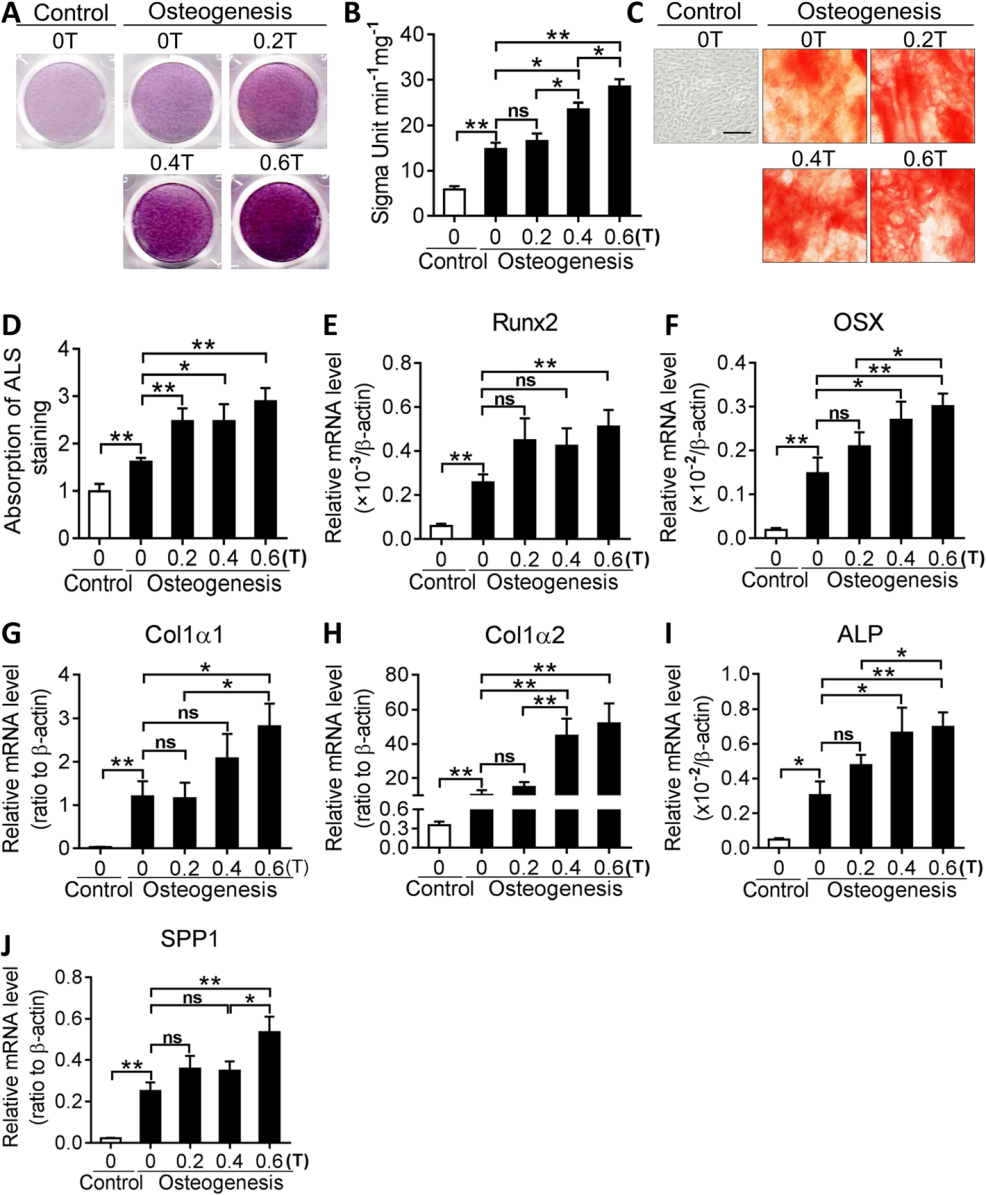
SMFs promote osteogenic differentiation of BMSCs. **a** Representative images of alkaline phosphatase (ALP) staining of BMSCs cultured in osteogenic medium and treated with gradient SMF for 3 days. Scale bar, 100 μm. **b** Detection of ALP activity. *P < 0.05, **P < 0.01 vs control or 0 T group. *n* = 4 per group. **c**, **d** Representative images of alizarin red S (ALS) staining (**c**) and quantification of matrix mineralization by ALS absorption (**d**) in BMSCs grown in osteogenesis induction medium and stimulated with gradient SMF for 21 days. Scale bar, 100 μm. *P < 0.05, **P < 0.01, as indicated. *n* = 4 per group. **e**–**j** Expression of key osteogenic transcription factors (*Runx2* and *Ost*) (**e** and **f**) and osteoblast marker genes (*Col1α1*, *Col1α2*, *Alp*, and *Spp1*) (**g**–**j**) in BMSCs grown in osteogenesis induction medium and treated with gradient SMF. *P < 0.05, **P < 0.01, as indicated. *n* = 8–10 per group. ns=not significant

To investigate whether exposure to SMF might affect the osteogenic differentiation of BMSCs, we examined the phenotypical changes of SMFs-challenged BMSCs cultured in osteogenesis induction medium. After osteogenic induction, we found that moderate SMFs enhanced the production of ALP, an early marker of osteogenesis in BMSCs in a dose-dependent manner (Fig. 4a, b). Consistently, ALS staining and quantification of ALS absorption revealed a significant enhancement of osteogenesis in SMF-stimulated BMSCs compared with controls (Fig. 4c, d). Moreover, the mRNA levels of the key osteogenic transcription factors *Runx2* and osterix (*Osx*) (Fig. 4e, f) and those of *col1α1*, *col1α2*, *Alp*, and *Spp1* osteogenic marker genes (Fig. 4g–j) were demonstrated to be increased in SMF-exposed BMSCs in a dose-dependent fashion. These results suggested that SMFs promoted osteoblast differentiation of BMSCs.

### Moderate SMFs facilitated BMSC differentiation to osteoblasts via activating the Runx2 signaling pathway

**Fig. 5:**
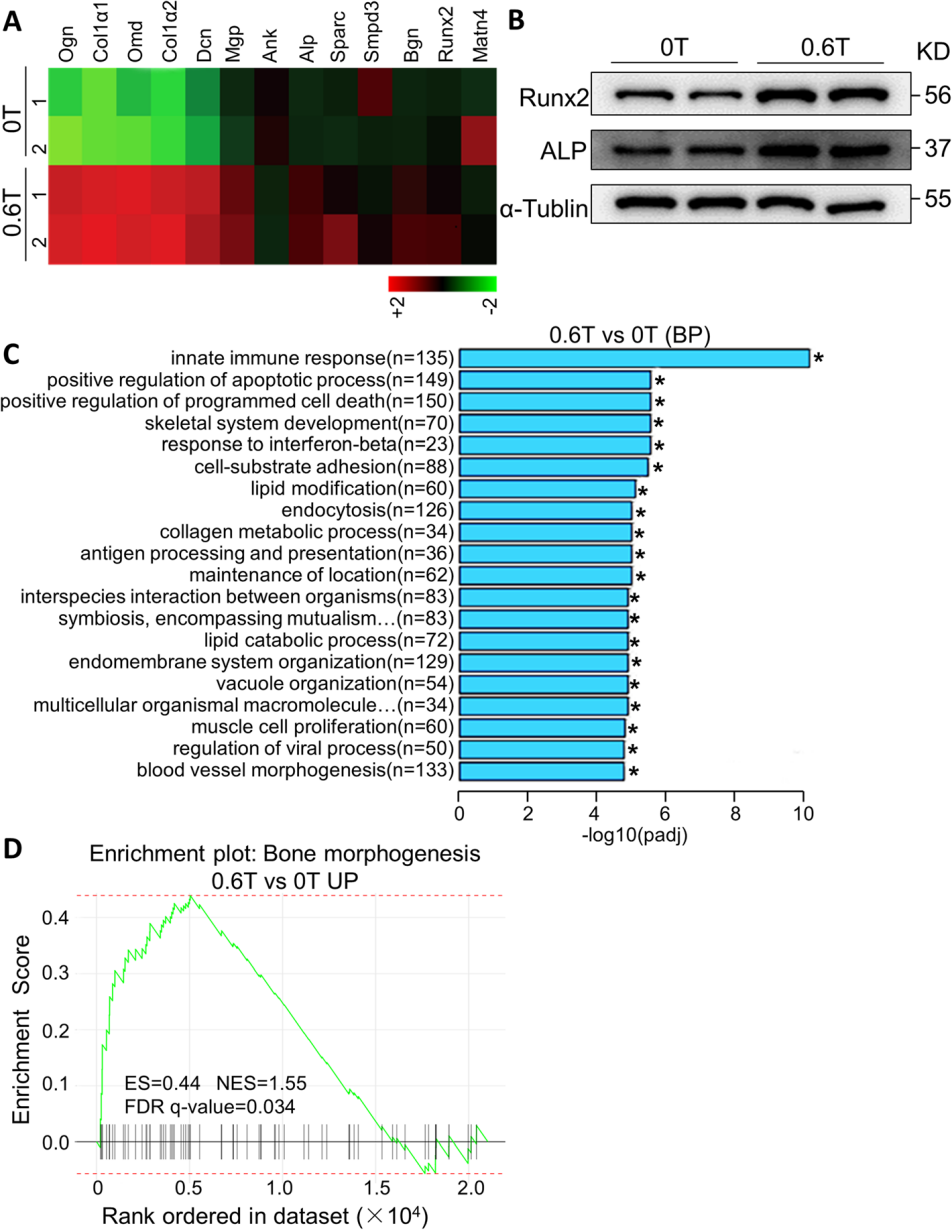
SMF activates RUNX2-mediated signaling in BMSCs in osteogenesis differentiation medium. **a** Heatmap of mRNA expression of osteogenic genes, as detected by deep sequencing, in BMSCs cultured in osteogenesis differentiation medium and stimulated with 0.6 T SMF for 48 h (fold change > 2, false discovery rate < 0.20). **b** Western blot analysis of RUNX2 and ALP in SMF-exposed BMSCs cultured in osteogenesis differentiation medium. **c** GO pathway enrichment analyses of significantly upregulated genes in SMF-exposed BMSCs grown in osteogenesis differentiation medium. Top 20 enriched pathways are shown.*P<0.05. **d** GSEA indicates that osteogenesis-positively correlated genes are significantly enriched in the SMF-exposed BMSCs compared with those in control cells

RNA-seq analysis uncovered that the osteogenic transcription factor *Runx2* and the osteoblast markers (*Col1α1*, *Col1α2*, *Alp*, *Ogn*, *Omd*, *Dcn*, *Mgp*, *Ank*, *Sparc*, *Smpd3*, *Bgn*, and *Matn4*) were also notably upregulated in SMF-exposed BMSCs cultured in osteogenesis induction medium, as compared with those in control BMSCs (Fig. 5a). Consistent with the mRNA expression results obtained by qPCR and RNA-seq, western blot analysis also showed an increase in the protein expression of the two key osteogenic markers, RUNX2 and ALP, in SMF-exposed BMSCs (Fig. 5b). Moreover, GO analysis showed that, upon osteogenic stimulation, SMF altered the gene expression associated with the development of the skeletal system, metabolic process of collagen, catabolic process of lipids, and others (Fig. 5c). Finally, GSEA analysis revealed that RUNX2 positively correlated genes were notably enriched in SMF-exposed BMSCs (0.6 T), compared with control cells (0 T) (FDR *q* value = 0.034) (Fig. 5d).

### Moderate SMFs reduced ATRA- or Dex-induced bone loss in mice

**Fig. 6:**
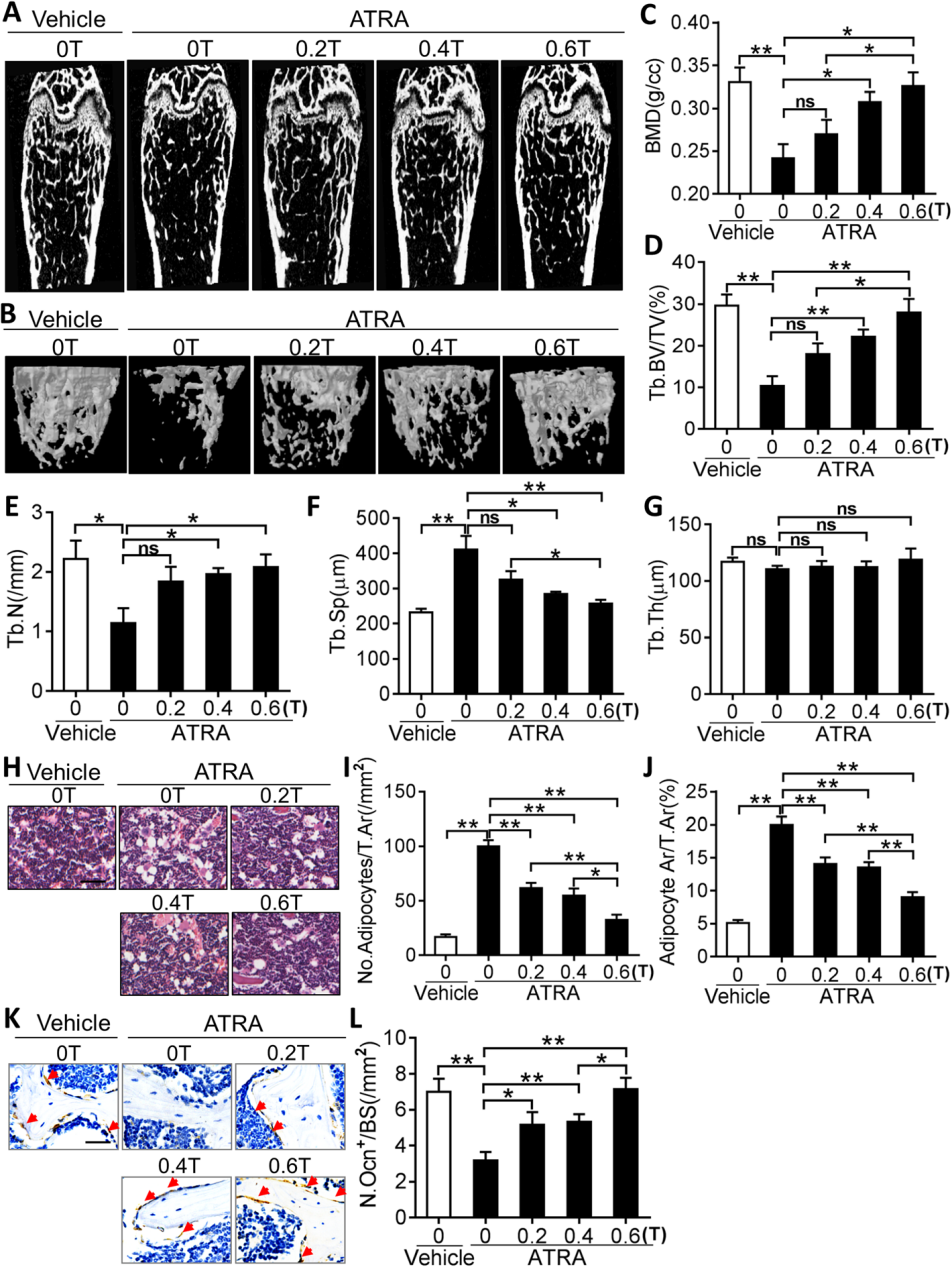
SMFs reduces all-trans retinoic acid (ATRA)-induced bone loss in mice. **a**, **b** Representative two-dimensional (2D) (**a**) and three-dimensional (3D) (**b**) microcomputed tomography (micro-CT) images of femurs from ATRA-treated mice with gradient SMF exposure for 21 days. **c**–**g** Quantitative micro-CT analysis of bone mineral density (BMD) (**c**), trabecular bone volume per tissue volume (Tb.BV/TV) (**d**), trabecular number (Tb.N) (**e**), trabecular separation (Tb.Sp) (**f**), and trabecular thickness (Tb.Th) (**g**) in SMF-exposed mice after ATRA injection. *P < 0.05, **P < 0.01, as indicated. *n* = 4–5 per group. **h**–**j** Representative images of H&E staining (**h**) with quantification of number (**i**) and area (**j**) of adipocytes in distal femurs. Scale bar, 100 μm. *n* = 5 per group. **k**–**l** Representative images of osteocalcin (OCN) immunohistochemical staining (**k**) with quantification of number (**l**) of osteoblasts in distal femurs. Scale bar, 100 μm. *P < 0.05, **P < 0.01, as indicated. *n* = 5 per group. ns=not significant

Chronic administration of the active metabolite of vitamin A, ATRA, has been reported to lead to significant bone loss in mice [28]. To explore whether SMFs might exert a preventive role in osteoporosis, ATRA-challenged mice were exposed to SMFs of varying intensities (0, 0.2, 0.4, and 0.6 T). ATRA significantly induced bone loss in mice by decreasing bone density (Fig. 6a–c) and Tb.BV/TV and Tb.N (Fig. 6d, e), thus increasing Tb.Sp (Fig. 6f) without markedly influencing trabecular thickness (Fig. 6g). Consistent with the micro-CT images (Fig. 6a, b), histological analysis showed that ATRA promoted the accumulation of adipocytes in the bone marrow (Fig. 6h–j) and reduced OCN-positive osteoblasts on both the trabecular and endosteal bone surfaces (Fig. 6k, l) in mice. However, SMFs were shown to notably attenuate the ATRA-induced bone loss in mice by increasing bone density and the total trabecular ratio, reducing the deposition of adipocytes in the bone marrow, and inducing osteoblast regeneration in mice (Fig. 6a–l). Moreover, this effect was noted to be occurring in an SMF intensity-dependent fashion.

**Fig. 7:**
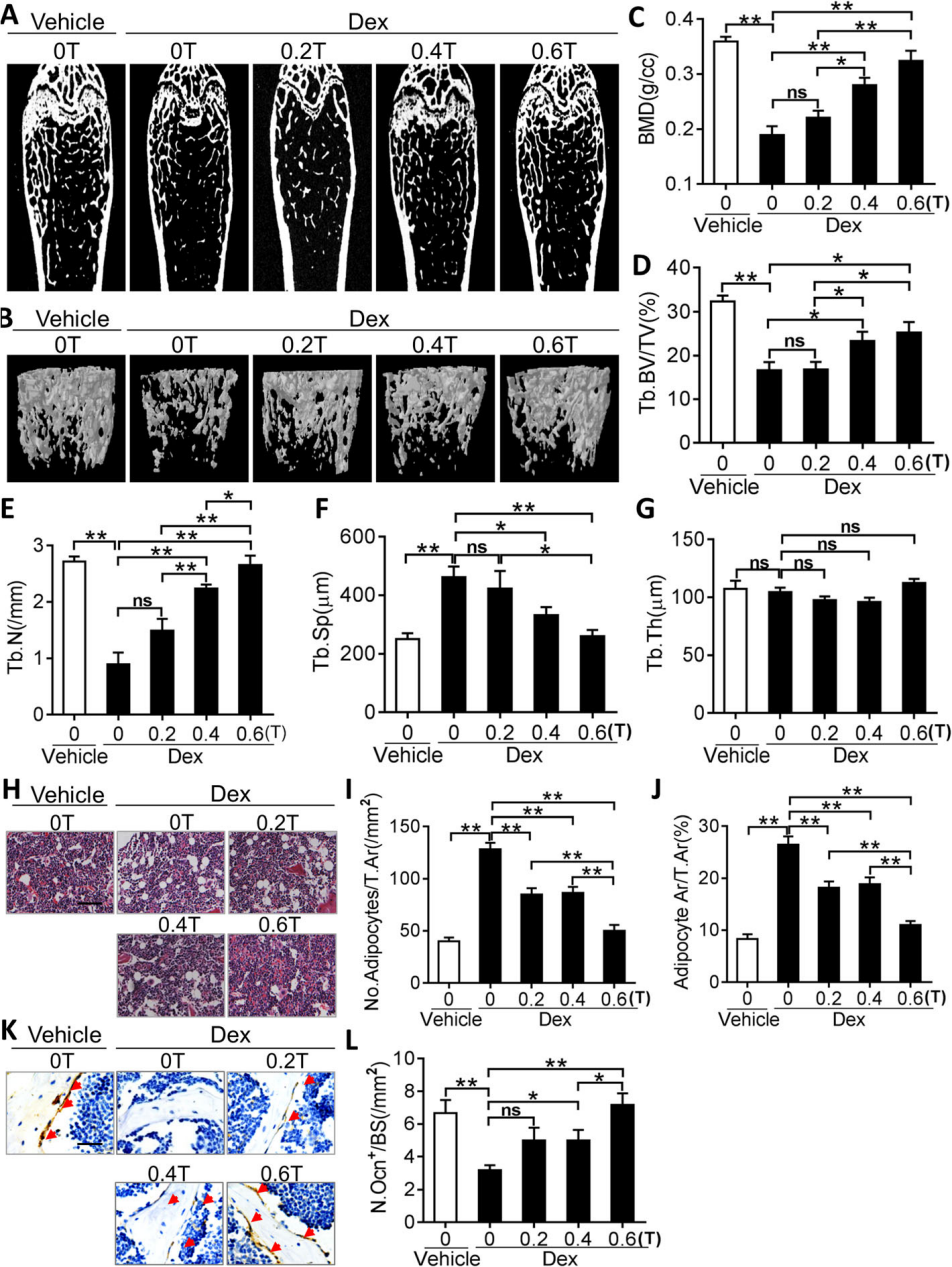
SMFs reduces dexamethasone (Dex)-stimulated osteoporosis in mice. **a**, **b** Representative 2D (**a**) and 3D (**b**) micro-CT images of femurs from control and Dex-treated mice with gradient SMF stimulation for 3 months. **c–g** Quantitative micro-CT analysis of BMD (**c**), BV/TV (**d**), Tb.N (**e**), Tb.Sp (**f**), and Tb.Th (**g**) in SMF-exposed mice after Dex injection. *P < 0.05, **P < 0.01, as indicated. *n* = 4–5 per group. **h**–**j** H&E staining (**h**) and quantification of number (**i**) and area (**j**) of adipocytes in distal femurs. Scale bar, 100 μm. *n* = 6 per group. **k**–**l** Representative images of OCN immunohistochemical staining (**k**) and quantification of number (**l**) of osteoblasts in distal femurs. Scale bar, 100 μm. *P < 0.05, **P < 0.01, as indicated. *n* = 5 per group. ns=not significant

We also tested the effects of SMF intervention on Dex-induced bone loss in mice. As shown in Fig. 7a–l, SMFs significantly prevented Dex-induced bone loss in mice by increasing bone density and the total trabecular ratio, reducing the deposition of adipocytes in the bone marrow and inducing osteoblast regeneration in mice in an intensity-dependent manner.

## Discussion

In this study, we stimulated BMSCs during adipogenic and osteogenic differentiation with different intensities of SMFs (0, 0.2, 0.4, and 0.6 T) and found that SMFs promoted osteoblastic differentiation but inhibited adipogenic differentiation of BMSCs in an intensity-dependent manner. Whole genomic RNA-seq analysis demonstrated that SMFs decreased PPARγ-driven gene expression but increased RUNX2-driven gene transcription in BMSCs. Moreover, SMFs markedly alleviated bone mass loss induced by either Dex or ATRA in mice. Thus, our study reveals the therapeutic potential of moderate-intensity SMFs in osteoporosis.

Magnetic fields are known to modulate the behavior of stem cells through multiple routes [31]. They are known to reduce inflammation, facilitate wound healing, and increase blood circulation [32]. In addition, magnetic field therapy has been approved for the management of pain and edema in superficial tissue inflammation by FDA [20]. Extremely low-frequency magnetic fields have been shown to influence cell proliferation by activation of Na^+^/K^+^ [33] and Ca^2+^ [34] channels. SMF has been commonly used in clinical practice as a tool, such as in magnetic resonance imaging (MRI). Moderate-intensity SMFs (1 mT to 1 T) were reported to enhance proliferation, migration, and dentinogenesis of dental pulp stem cells by activating the p38 mitogen-activated protein kinase pathway [35–37] and to induce osteo/odontogenesis and mineralization in dental pulp stem cells [37, 38]. Similarly, moderate-intensity SMF also promoted neuronal differentiation in fetal rat brain neural progenitor cells [39] and induced the proliferation and osteoblastic differentiation of BMSCs [40, 41].

BMSCs are known to differentiate into adipocytes instead of osteoblasts with aging, leading to the occurrence of osteoporosis, which is characterized by bone loss and progressive accumulation of fat [42, 43]. Here, we demonstrated that SMFs repressed adipogenic differentiation but promoted osteogenic differentiation of BMSCs in an intensity-dependent manner by suppressing the PPARγ signaling pathway and activating the RUNX2 signaling pathway. Their capacity to differentiate into osteoblastic and adipogenic lineages is dependent on various signaling pathways and key transcription factors [44]. Activation of *Runx2* and *Osx* has been found to promote osteogenic differentiation and inhibit adipogenic differentiation of BMSCs [45]. Conversely, *Cebpα*, *Cebpβ*, and *Pparγ* have been reported to drive adipogenesis and disrupt osteogenic differentiation of BMSCs [46, 47]. We demonstrated that moderate-intensity SMFs notably inhibited the expression of the *Cebpβ* and *Pparγ* adipogenic transcription factors in BMSCs cultured in adipogenesis induction medium but upregulated the *Runx2* osteogenic transcription factor and its downstream targeted genes in BMSCs upon osteogenic stimulation. Bioinformatics analysis showed that SMFs altered the gene expression associated with the metabolism of fat in adipogenic medium-cultured BMSCs and promoted the expression of genes associated with skeletal development and osteoblast differentiation in osteogenic medium-cultured BMSCs.

Because SMFs are time-independent fields whose intensity can be spatially dependent, they are able to freely penetrate the biological tissues [48]. The biological effects of SMFs are known to be related to the dosing regimen, target tissue, magnet characteristics, and the magnet support device [49]. Mechanistically, SMF has been shown to be able to directly regulate the cell shape and plasma membrane structure [50], interact with magnetic materials found in tissues [51], and modulate intracellular levels of reactive oxygen/nitrogen species [52]. Additionally, SMFs have also been reported to exert their effects by enhancing synthesis and secretion of membrane-derived microvesicles (MVs), mediating drug delivery and perhaps by inducing mitophagy [31, 53, 54]. Recently, an iron-sulfur cluster protein, called iron-sulfur cluster assembly protein 1, was identified and validated in mammalian cells as a magnetic sensor [55, 56]. However, further studies are required to investigate how SMFs regulate the RUNX2/PPARγ-mediated signaling pathways during BMSC differentiation.

Glucocorticoids are widely prescribed for the treatment of autoimmune and inflammatory diseases; however, long-term treatment in the clinical setting has been shown to frequently cause osteoporosis. We also showed here that moderate SMFs promoted BMSC differentiation to osteoblasts and prevented ATRA- and Dex-induced bone loss in mice. Moreover, others have also reported that SMFs markedly accelerate the healing of osteotomized femur and promote bone regeneration in rats [23, 57]. Therefore, SMF might be helpful for the prevention and treatment of osteoporosis.

Both SMF and electromagnetic fields (EMF) including pulsed electromagnetic fields (PEMF) can substantially facilitate bone healing. For instance, EMFs positively regulates osteogenic lineage commitment of BMSCs [58]. Similarly, low-frequency PEMFs facilitate bone repair by promoting osteoblast proliferation and osteogenic differentiation of BMSCs [59]. Thus, as a noninvasive, effective, and clinical safety treatment, EMF therapies have been commercially used to promote bone un-united fracture healing [59–62] and other skeletal disorders [63]. Compared to EMFs, SMFs appear more convenient without additional electrical devices, which avoids electric or heat hazard to surrounding tissue [64]. Importantly, SMF stimulation is suitable for long-term topical treatment [40, 65]. In this study, we demonstrate the moderate-intensity SMFs ranging from 0.2 T to 0.6 T induce bone repair in mice in a dose-dependent manner.

## Conclusions

In summary, SMFs protected against ATRA- or Dex-induced osteoporosis in mice by promoting the RUNX2-mediated osteogenic differentiation and suppressing the PPARγ-mediated lipogenic differentiation of BMSCs. These results suggested that SMF-based magnetotherapy might be beneficial for patients with osteoporosis.

## Highlights

- Moderate-intensity SMF promotes osteogenic differentiation of BMSCs.
- Moderate-intensity SMF suppresses lipogenic differentiation of BMSCs.
- Moderate-intensity SMF reduces bone mass loss induced by either dexamethasone or all-trans retinoic acid in mice.

## Supplementary Information

**Additional file 1:** **sFig. 1.** The identification of BMSCs by flow cytometry.

**Additional file 2:** **Supplementary Table 1.** Real time-PCR primer sequences.

## Data Availability

All data needed to evaluate the conclusions in the paper are present in the paper and/or the Supplementary Materials. Additional data related to this paper may be requested from the authors.

## Abbreviations

ALS: Alizarin red S
ALP: Alkaline phosphatase
ATRA: All-trans retinoic acid
ASCs: Adipose-derived stem cells
BMSCs: Bone marrow-derived mesenchymal stem cells
BMD: Bone mineral density
Cd36: Cluster of differentiation 36
Dex: Dexamethasone
Fabp4: Fatty acid binding protein 4
GO: Gene ontology
GSEA: Gene-set enrichment analysis
ORO: Oil red O
OCN: Osteocalcin
Osx: Osterix
SMFs: Static magnetic fields
PEMF: Pulsed electromagnetic fields
Tb.BV/TV: Trabecular bone volume per tissue volume
Tb.N: Trabecular number
Tb.Sp: Trabecular separation
