# Supplementary figures and images for: Moderate SMFs attenuate bone loss in mice by promoting directional osteogenic differentiation of BMSCs
Source: Stem Cell Res Ther. 2020 Nov 16;11:487. doi: 10.1186/s13287-020-02004-y (PMC7667787; doi:10.1186/s13287-020-02004-y)

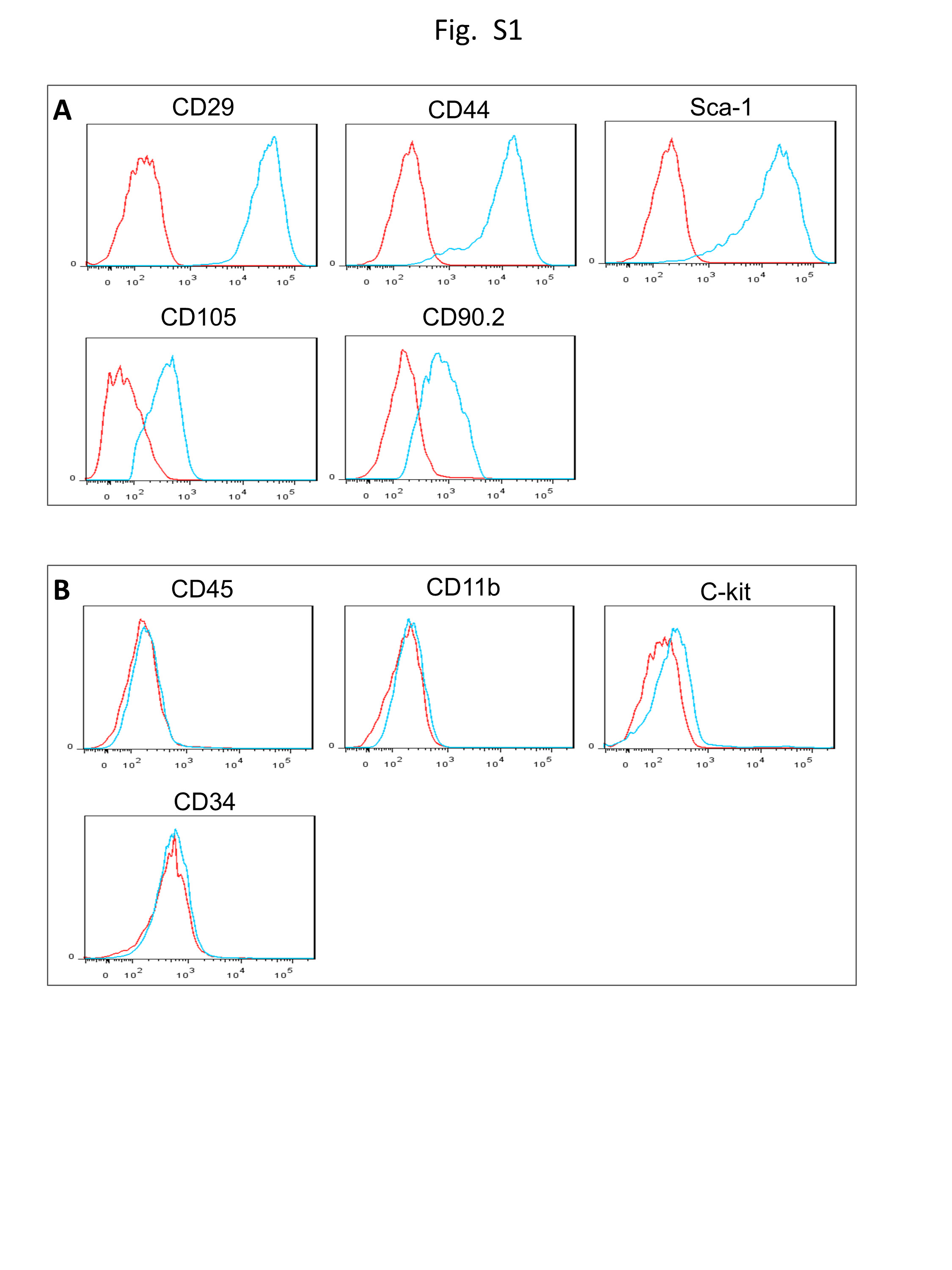

Supplement: Supplementary file 1 — Additional file 1: sFig. 1. The identification of BMSCs by flow cytometry. [file 13287_2020_2004_MOESM1_ESM.tif]
