# Supplementary material for: Moderate SMFs attenuate bone loss in mice by promoting directional osteogenic differentiation of BMSCs
Source: Stem Cell Res Ther. 2020 Nov 16;11:487. doi: 10.1186/s13287-020-02004-y (PMC7667787; doi:10.1186/s13287-020-02004-y)
Supplement: Supplementary file 2 — Additional file 2: Supplementary Table 1. Real time-PCR primer sequences. [file 13287_2020_2004_MOESM2_ESM.docx]

**Supplemental Table 1.**

**Real time-PCR primer sequences**

| **Gene** | **Forward (5’ to 3’)** | **Reverse (5’ to 3’)** |
| --- | --- | --- |
| Cebpα  Cebpβ  Cebpδ  PPARγ  Adiponectin  CD36  Fabp4  Runx2  Osterix  ALP  SPP1  Col1α1  Col1α2  β-actin | GCGGGAACGCAACAACATC  AGCCCCTACCTGGAGCCGCTCGCG  CGACTTCAGCGCCTACATTGA  TCGCTGATGCACTGCCTATG  TGTTCCTCTTAATCCTGCCCA  ATGGGCTGTGATCGGAACTG  AAGGTGAAGAGCATCATAACCCT  CCAACCGAGTCATTTAAGGCT  ATGGCGTCCTCTCTGCTTG  CCAACTCTTTTGTGCCAGAGA  AGCAAGAAACTCTTCCAAGCAA  GCTCCTCTTAGGGGCCACT  CAACTCAGCTCGCCTTCATG  GTACCACCATGTACCCAGGC | GTCACTGGTCAACTCCAGCAC  GCGCAGGGCGAACGGGAAACCG  CTAGCGACAGACCCCACAC  GAGAGGTCCACAGAGCTGATT  CCAACCTGCACAAGTTCCCTT  GTCTTCCCAATAAGCATGTCTCC  TCACGCCTTTCATAACACATTCC  GCTCACGTCGCTCATCTTG  TGAAAGGTCAGCGTATGGCTT  GGCTACATTGGTGTTGAGCTTTT  GTGAGATTCGTCAGATTCATCCG  CCACGTCTCACCATTGGGG  AGGTACGCAATGCTGTTCTTG  AACGCAGCTCAGTAACAGTCC |
